# Systematizing peer recovery support services for substance use disorder: a taxonomy for measuring recovery milestones

**DOI:** 10.3389/fpubh.2025.1529078

**Published:** 2025-05-12

**Authors:** Kimberly Horn, Ryan E. Flinn, Angela Marie Hagaman, Kristyn Zajac, Lauren A. Hoffman, Melissa N. Poulsen, Camille C. Cioffi, Ducel Jean-Berluche, Ethan Spana, Patrick F. Hibbard, Tess K. Drazdowski, Aaron Hogue

**Affiliations:** ^1^Institute of Policy and Governance, Virginia Tech, Blacksburg, VA, United States; ^2^Department of Education, Health & Behavior Studies, University of North Dakota, Grand Forks, ND, United States; ^3^Addiction Science Center, College of Public Health, East Tennessee State University, Johnson City, TN, United States; ^4^Calhoun Cardiology Center, University of Connecticut School of Medicine, Farmington, CT, United States; ^5^Recovery Research Institute, Center for Addiction Medicine, Massachusetts General Hospital and Harvard Medical School, Boston, MA, United States; ^6^Department of Population Health Sciences, Geisinger, Danville, PA, United States; ^7^Prevention Science Institute, University of Oregon, Eugene, OR, United States; ^8^Be Well Institute for Substance Use and Related Disorders, University of Texas Health Science Center at San Antonio, San Antonio, TX, United States; ^9^Chestnut Health Systems – Lighthouse Institute, Chicago, IL, United States; ^10^Partnership to End Addiction, New York, NY, United States

**Keywords:** substance use, peers, recovery support services, peer recovery support specialists, taxonomy

## Abstract

**Background:**

Recovery from substance use disorder (SUD) is a complex and individualized process requiring multifaceted support systems. Peer recovery support services (PRSS), provided by Peer Workers, bridge the gap between formal intervention and personal recovery experiences. Drawing on shared lived experience, Peer Workers offer essential support to fellow Peers navigating recovery. However, variability in PRSS roles, training, and settings creates challenges for consistent evaluation and measurement of effectiveness.

**Objective:**

Introduce a systematic taxonomy to clarify the roles, functions, and activities within PRSS, providing a structured framework for evaluating their impact on key SUD recovery milestones.

**Methods:**

The taxonomy was developed through a rapid narrative literature review, expert consultation, and an iterative consensus process informed by a Delphi-like approach. A multidisciplinary task group of PRSS scientists and practitioners, SUD treatment providers, and individuals in recovery contributed to its refinement. The framework aligns with key components from SAMHSA’s national standards (SAMHSA, 2023) to enhance consistency across practice settings.

**Results:**

Comprising six primary taxons and 20 branches, the taxonomy organizes PRSS components into structured categories. It classifies variations in lived experience (e.g., direct, indirect, and hybrid), training levels (e.g., basic, specialized, continuous education, and formal education), support approaches (e.g., Peer Worker-led services), and support settings (e.g., community-based, clinical, and justice system). Additionally, it categorizes peer support activities into four core domains: emotional, informational, instrumental, and affiliational support. The taxonomy integrates a structured model for PRSS evaluation, identifying mediators (e.g., support approaches) and moderators (e.g., training levels) that influence recovery outcomes.

**Conclusion:**

The proposed taxonomy and integrated evaluation model provide a standardized framework for researchers and practitioners to systematically assess PRSS impact on recovery milestones. By establishing a common language, the taxonomy enhances consistency in PRSS research, identifies empirically supported peer support practices, and informs targeted training and strategic implementation. Future research should prioritize empirical testing of this framework to refine its applicability across diverse PRSS settings and enhance intervention effectiveness and scalability.

## Introduction

SUD represents a major public health concern. In 2022, nearly 49 million US individuals aged 12 or older reported having an SUD within the past year, including 29.5 million with an alcohol use disorder (AUD), 27.2 million with a drug use disorder (DUD), and almost 8 million with both AUD and DUD (4). SUD involves functional changes to brain circuits related to reward, stress, and self-control (1), and are associated with physical, psychological, and social comorbidities that contribute to shorter life expectancy (2). Between 2019 and 2022, drug overdose deaths in the U.S. rose significantly, with an estimated 107,941 overdose fatalities reported in 2022 (3). Although national data from 2023 suggest an overall 3% decline in drug overdose rates, some states reported increases, and overdose deaths from substances like cocaine and methamphetamine continued to rise (4).

The use of PRSS is increasingly common across the continuum of SUD care (5). These services support key SAMHSA (6) recovery principles by engaging individuals in SUD treatment, assisting with transitions across levels of care, helping to secure employment and housing, linking to community-based support services and mutual-help organizations, addressing criminal justice issues, and educating about illness management (7). The delivery of these services by PRSS, which may at times include conventional case management support (8) help clients benefit from these services while reducing stigma (9). PRSS occur in a variety of settings, including recovery housing, shelters, emergency rooms, inpatient and outpatient programs, primary care, courts, correctional facilities, colleges, community spaces, and recovery high schools (10).

Recovering from substance use disorder (SUD) is a dynamic, complex process requiring multifaceted support systems. Peer recovery support services (PRSS) bridge the gap between formal intervention and the personal experience of recovery, with “Peer Workers” employed specifically to leverage their lived experience to support “Peers” facing SUD recovery. The Substance Abuse and Mental Health Service Administration (6) defines recovery as a process of change in which individuals improve their health and wellness, lead a self-directed life, and strive to reach their full potential. Many people recovering from SUDs require various approaches, tools, and support systems. One such approach is assistance from peer recovery support services (PRSS). Peer recovery support services (PRSS) bridge the gap between formal intervention and the personal experience of recovery, with “Peer Workers” employed specifically to leverage their lived experience to support “Peers” facing SUD recovery, serving as a critical component in the recovery process (11). PRSS provides a non-clinical approach led by individuals with lived experience in similar conditions to help initiate, pursue, and sustain long-term recovery from SUD and mental health challenges (7, 12).

Although PRSS research is in its infancy, studies show evidence of PRSS efficacy across certain settings and outcomes (13, 14). A systematic review revealed that PRSS models contribute to reductions in substance use and relapse rates and improve treatment retention and satisfaction. However, methodological inconsistencies (e.g., poorly defined roles and procedures) and null results limit findings, indicating the need for additional research.

The rapid expansion of PRSS underscores the need for a framework that clearly delineates the essential elements of these services to measure their impact on recovery milestones. Currently, the PRSS field lacks a **taxonomy**—a structured classification system that organizes information into categories based on shared characteristics, simplifying complex data for analysis and understanding. Although SAMHSA and the Peer Recovery Center of Excellence (5) established guidelines for PRSS delivery, little is known about the specific contexts and conditions where PRSS interventions are most effective. Additionally, the absence of standardized terms for job titles, roles, activities, and settings creates barriers to understanding the full impact of Peer Workers (12, 15–17). As more funded studies rigorously evaluate PRSS across settings, the lack of a common nomenclature continues to limit communication within the research community and hinders scientific and clinical progress.

Building on an existing taxonomy for linkage facilitation services for opioid use disorder (18), the present taxonomy categorizes key factors—Lived Experience, Training, Support Approach, Support Settings, Support Activities, and Recovery Milestones —into empirically driven taxons and branches. The end goal is informing and enhancing PRSS effectiveness through structured, consistent nomenclature, measurement, and evaluation.

Specifically, this article presents a taxonomy to support future PRSS studies by:

1. **Establishing a common language**: Offering standardized and consistent terminology for research, facilitating clear communication and comparison of findings.
2. **Identifying impactful roles and functions**: Defining effective roles, functions, and activities that drive progress toward recovery milestones.
3. **Customizing PRSS interventions**: Enabling tailored methods that align with individual recovery trajectories, recognizing that effective support must be adaptable.
4. **Refining training and strategy implementation**: Providing targeted recommendations to optimize training, support mechanisms, and overall strategy, ensuring that Peer Workers are adequately prepared and interventions are evidence-based.
5. **Clarifying the influence of lived experience**: Investigating the contexts under which lived experience (direct, indirect, or hybrid) is most effective, providing insights to optimize different subgroups within the recovery population.

Our approach is unique in that it integrates a PRSS taxonomy framework to identify variables which will be useful to include in evaluating the impacts of Peer Workers on peers’ substance use recovery milestones, including the possible mechanisms by which change occurs. Drawing from a diverse panel of experts—including scientists, practitioners, and individuals with lived experience—the framework ensures a meaningful and practical taxonomy applicable across various contexts. By identifying potential mediators (such as specific strategies or settings) and moderators (such as training levels), our model recommends nuanced examinations of conditions under which PRSS and Peer Workers are most effective. Going a step beyond typical taxonomy categorization, we provide a foundation for empirical testing and iterative refinement of a framework adaptable to diverse populations and recovery settings.

PRSS complements but does not replace clinical treatment. Unlike medical or therapeutic interventions, PRSS is rooted in peer-driven engagement, non-clinical mentorship, and shared lived experience. This taxonomy categorizes the key functions of PRSS while recognizing its distinct role within the broader recovery ecosystem. Ultimately, the taxonomy is designed to benefit multiple audiences: (1) researchers by providing a structured framework for PRSS evaluation, (2) practitioners by standardizing peer worker roles and functions, and (3) policymakers by informing training, certification, and workforce development standards.

## Methods

The authors comprise a sub-group of an expert panel formed from the Peer Recovery Support Special Interest Group within The Consortium on Addiction Recovery Science (CoARS, 3R24DA051946-01S1). This initiative is funded by the National Institute on Drug Abuse (NIDA) and is coordinated by Dr. Aaron Hogue (Principal Investigator). When the article concept began, the panel comprised nearly two dozen scientists from universities and private research centers and other experts on PRSS, including SUD treatment providers, and individuals with lived SUD recovery experience. Together, they provided a diverse and comprehensive perspective to the development of this taxonomy, aimed at advancing the effectiveness and evaluation of PRSS interventions.

Our methods did not employ formal interviews or focus groups, but we incorporated elements of qualitative rigor aligned with COREQ principles (19) to enhance transparency and reliability in the taxonomy development process. Specifically, structured feedback from experts and iterative consensus-building were key methodological components. While COREQ is primarily designed for studies involving interviews, our approach relied on structured expert feedback, literature synthesis, and a Delphi-like process to ensure rigor.

## Taxonomy development

### Literature review

We conducted a targeted, narrative literature review to identify key PRSS components, rather than an exhaustive systematic review. This literature review served to identify key constructs rather than exhaustively map all PRSS studies. This approach focused on identifying exemplars and case studies relevant to each taxon. Searches were performed in PubMed, PsycINFO, and Scopus using terms such as ‘peer recovery support services,’ ‘recovery milestones,’ and ‘substance use disorder treatment.’ We then used these studies to inform our taxonomy by illustrating where PRSS practices align with existing research. These study references provided empirical justification for inclusion and helped shape the classification structure. In cases where literature was limited, expert consensus filled the gap by integrating best practices from PRSS implementation.

The aim was to provide a balanced, accessible overview to broadly understand the recovery milestones commonly associated with successful SUD treatment and the role of PRSS in fostering these recovery milestones. In the end, this approach allowed us to synthesize existing research and highlight examples of PRSS implementation for each taxon while identifying gaps in the current understanding of PRSS practices and their effectiveness.

### Expert consultation

To gain deeper insights into the practical aspects of PRSS, we convened our panel of CoARS experts within the PRSS Special Interest Group, comprising PRSS practitioners, SUD treatment providers, and individuals with lived recovery experience. The expert panel’s input was essential in shaping the preliminary structure of the taxonomy.

### Iterative expert consensus approach

We employed an iterative consensus approach among our expert panel to reach agreement on the taxonomy’s content and structure, including the final six taxons and branches. The multidisciplinary group engaged in regular structured monthly Zoom meetings over a 8-month time period and ad-hoc email discussions among authors, as needed, providing critical perspectives on key elements of peer support and recovery milestones. To manage dissent and lack of agreement, we implemented an iterative feedback cycle, allowing panelists to provide subjective ratings, comments, and modifications at each stage. When consensus was not reached on a given component, we incorporated additional rounds of discussion and adjusted definitions until alignment was achieved.

Experts ranked the importance and relevance of various PRSS components and recovery milestones, and successive rounds of input over several months enabled refinement of the taxonomy. This approach ensured the taxonomy was comprehensive, applicable, and aligned with current practices (20, 21).

### Field responses

Preliminary versions of the taxonomy were vetted and discussed with active PRSS practitioners to validate applicability in real-world settings. Practitioners provided valuable observations and feedback, which informed practical adjustments to the taxonomy. This step was crucial in refining the model to align with the everyday realities and needs of PRSS Peer Workers and peers.

## Expert panel results summarized

The taxonomy evolved through a structured consensus process. Initially, our team synthesized themes from the literature review. These themes were presented and discussed among the expert panel, who refined and reorganized them based on practical application and research alignment. Specific refinements included the differentiation of lived experience categories, the restructuring of support activities, and the expansion of training types. Successive rounds of feedback incorporated expert revisions and field practitioners further validated the framework by assessing real-world applicability, ensuring that all components aligned with PRSS delivery practices.

The end goal was to develop a taxonomy that serves as a comprehensive framework for evaluating the most impactful PRSS roles and functions. This framework provides valuable insights into individualized recovery approaches and guides targeted recommendations for enhancing PRSS training methodologies and optimizing strategic implementation.

To reiterate, in this taxonomy “Peer Worker/s” refers to individuals with lived experience (direct, indirect, or hybrid) employed to provide PRSS to individuals with SUD (18), sometimes referred to as “Clients.” Many Peer Workers, however, prefer the ideological term “peers.” Herein, the taxonomy uses the terms “Peer Workers” and “peers.”

The present taxonomy organizes PRSS components into **six primary taxons, a-f**: a. Lived Experience, b. Training, c. Support Approach, d. Support Settings, e. Support Activities, and f. Recovery Milestones. Empirically-based, each taxon comprises distinct branches and sub-branches that address the diversity and complexity of PRSS roles. **Lived Experience** includes branches such as direct, indirect, hybrid, and remote experience, with sub-branches capturing specific types within these categories. **Training** covers basic, specialized, continuous education, and formal certification, with sub-branches detailing areas relevant to PRSS delivery. **Support Approach** identifies primary delivery methods, while **Support Settings** encompasses various environments in which PRSS operates. **Support Activities** is organized by emotional, informational, instrumental, and affiliational support types, and **Recovery Milestones** categorizes proximal and distal recovery milestones. Altogether, the taxonomy comprises **six taxons, 20 branches, and 87 sub-branches**, providing a detailed structure for systematically evaluating PRSS practices and measuring their outcomes across various recovery contexts.

## Taxonomy of peer recovery support services

### a. Peer worker lived experience

SAMHSA (22) (p. 12) defines lived experience as: “personal knowledge about the world gained through direct, first-hand involvement in everyday events rather than through representations constructed by other people.” PRSS, therefore, represents a constellation of “Peer Worker-based mentoring, education, and support service provided by individuals in recovery from substance use disorders to individuals with substance use disorders or co-occurring substance use and mental disorders” (11) (p. 853). Peer Workers agree that a shared lived experience of the concerns to be addressed through PRSS is considered foundational to their effectiveness and distinguishes them from providers belonging to other professions that provide behavioral health or medical services (23).

Peer Workers may also simultaneously volunteer their time in unpaid helping roles (e.g., 12 Step Sponsor) (24), which they may consider to be part of their own personal recovery maintenance. They describe that providing peer support to others also strengthened their own recoveries and sense of accomplishment, suggesting that a primary premise of peer support (mutual benefit) is experienced by at least some Peer Workers employed in this role (25). Peer Workers identify in workplaces, organizations, and states by a variety of titles (e.g., recovery coach, peer support specialist, certified peer recovery support specialist) (23).

#### a1. Direct lived experience

*Boundary/operational definition*: Peer workers who have personal lived experience of a substance use (i.e., personal diagnosis or problem) and have navigated the recovery process, which directly informs their peer support role.

- **a1.1. Substance use disorder (SUD) experience**: Peer Workers have a personal history of substance use disorder (SUD) or a substance use problem without a formal diagnosis, providing them with firsthand knowledge and experience of the recovery process.
- **a1.2. Dual diagnosis experience**: Peer Workers who have experienced both SUD and a co-occurring mental health disorder, giving them insights into the challenges of managing multiple conditions.
- **a1.3. Long-term recovery experience**: Peer Workers who have maintained sustained recovery over an extended period, offering perspectives on maintaining long-term sobriety and well-being.

#### a2. Indirect lived experience

*Boundary/operational definition*: Individuals who have been significantly involved in the recovery journey of others or have professional exposure to recovery, offering a supportive perspective without personal SUD experience.

- **a2.1. Family/close relationship**: Individuals who have supported a family member or close friend through SUD recovery, providing insights into the familial impact of addiction and recovery.
- **a2.2. Community involvement**: Individuals who are deeply involved in community recovery initiatives or advocacy but have not personally experienced SUD.

#### a3. Hybrid lived experience

*Boundary/operational definition*: Individuals who combine personal and professional experiences, or those with a multigenerational or cultural perspective, enriching their peer support roles.

- **a3.1. Combined personal and professional experience**: Peer Workers who have both personal experience with SUD and professional involvement (employment) in providing recovery support services.
- **a3.2. Multigenerational experience**: Individuals who have experienced SUD recovery across multiple generations within their family, providing a broader perspective on the impact of SUD.
- **a3.3. Cultural/community-based experience**: Peer Workers who not only have personal recovery experience but also deep engagement with cultural or community recovery practices.

### b. Training

In the United States, Peer Worker training and certification occur at the state level, resulting in considerable variation. The Peer Recovery Center of Excellence (2022) notes that states may certify Peer Workers for substance use recovery, mental health recovery, or both through integrated certification programs. Certification may be administered by state agencies, third-party organizations, or both. Requirements for lived experience also differ; some states restrict certification to those with direct lived experience, while others accept candidates with indirect lived experience. Additionally, some states mandate a period of abstinence from substances before certification, while others do not. Educational requirements typically involve 40–46 h of training, with most states requiring an examination for certification. To address these inconsistencies, SAMHSA’s (26) National Model Standards recommend a comprehensive curriculum covering core competencies such as diversity, PRSS roles, recovery principles, trauma-informed care, and crisis management. These standards also emphasize advocacy, ethics, and self-care to ensure that Peer Workers are thoroughly prepared. Training further includes 12 core competencies for PRSS, focusing on peer relationships, recovery planning, crisis management, and communication skills to promote collaboration and leadership in recovery support [SAMHSA], 2015.

#### b1. Basic training

*Boundary/operational definition*: Foundational training for PRSS equips Peer Workers with essential skills and knowledge to support recovery in various settings (27).

- **b1.1. Introduction to peer support**: Covers fundamental principles, ethics, and roles of peer support within the recovery process, emphasizing the value of lived experience, mutual support, and the role of Peer Workers as facilitators of change.
- **b1.2. Communication skills**: Training includes effective communication techniques such as active listening, empathy, non-verbal cues, and conflict resolution skills, which are essential for building trust and rapport with peers.
- **b1.3. Boundaries and ethics**: Instruction focuses on maintaining professional boundaries, understanding ethical considerations in peer support, and navigating the dual relationship dynamics that may arise given the shared lived experience.

#### b2. Specialized training

*Boundary/operational definition*: Advanced training focused on specific areas of peer support, addressing challenges or needs of peers in recovery and Peer Workers’ self-identified training needs (28–31).

- **b2.1. Mental health support training**: Specialized training in supporting Peer Workers with co-occurring mental health conditions, ensuring comprehensive care.
- **b2.2. Substance use disorder (SUD) support training**: Advanced knowledge in SUD-specific interventions and support strategies tailored to those in recovery.
- **b2.3. Trauma-informed care**: Training that emphasizes the importance of understanding and addressing trauma within the recovery process.
- **b2.4. Crisis intervention**: Preparation for handling crisis situations, such as overdoses or severe mental health episodes, in a peer support context.
- **b.2.5 Setting or population specific training**: Training that focuses on non-traditional settings providing peer support such as primary care or law enforcement co-response teams and/or training specific to certain sub-populations such as victims of sexual violence or trafficking.
- **b.2.6. Peer worker specialist certification**: State or nationally recognized certifications specifically for peer support roles, often involving a structured curriculum and examination.

#### b3. Continuous education

*Boundary/operational definition*: Ongoing training and professional development that ensures Peer Workers remain up-to-date with best practices and emerging trends in recovery support.

- **b3.1. Advanced certifications**: Further certifications that build on initial training, offering specialized expertise in areas such as recovery coach supervision or trauma-informed care.
- **b3.2. Workshops and seminars**: Regular participation in educational sessions on current issues, techniques, and research in peer support and recovery.
- **b3.3. Reflective practice**: Engaging in self-reflection, supervision, or review to continually assess and improve Peer Worker support practices.

#### b4. Formal education and certification types

*Boundary/operational definition*: Educational levels or formal certifications that potentially influence Peer Workers’ qualifications or their role and effectiveness in providing peer support. *Note*: Although formal education is not required for PRSS certification, several states mandate it for Medicaid billing and reimbursement in certain roles (22). Therefore, measuring educational attainment could be useful, especially in contexts where it directly impacts billing and service delivery.

- **b4.1. High school diploma/GED**: The foundational educational level required for *some* Peer Worker support roles, in certain states.
- **b4.2. Some college/associate degree**: Partial or full completion of an associate’s degree, which may include coursework related to psychology, social work, or addiction studies.
- **b4.3. Bachelor’s degree or higher**: Completion of a bachelor’s degree or more advanced education in relevant fields (e.g., social work, counseling), potentially offering deeper knowledge and broader opportunities in Peer Worker support roles.

### c. Support approach

As described previously, Peer Workers possess and share with peers their own lived experience with substance use problems and recovery, including relevant lived experience in navigating multiple systems that interface with peers living with SUD. This explicit sharing of their own lived experience and leveraging of lessons learned to support others in their recovery is the defining characteristic of Peer Workers, distinguishing them from other professions (23). Peer Workers provide multiple types of support to peers across phases of SUD treatment (32) and may also be leveraged in various prevention, health promotion, and linkage services (11, 18).

#### c1. Peer worker-delivered self-help

*Boundary/operational definition*: Informal, Peer Worker-facilitated support where Peer Workers lead groups or provide individual guidance based on shared experiences.

- **c1.1. Group facilitation**: Leading or facilitating peer support groups, often in community settings, to foster mutual support.
- **c1.2. One-on-one peer mentorship**: Providing direct, individual support and guidance tailored to the peer’s unique recovery journey.
- **c1.3. Informal social support networks**: Creating and maintaining networks, both in-person and online, provided by peer workers offering ongoing social support.

#### c2. Peer worker-run services

*Boundary/operational definition*: Structured programs or services that are entirely operated by Peer Workers, emphasizing the principles of mutual support and self-help.

- **c2.1. Recovery coaching**: Providing structured guidance and goal-setting to Peer Workers, focusing on achieving and maintaining recovery milestones.
- **c2.2. Peer worker-led workshops**: Educational sessions led by Peer Workers, covering topics such as relapse prevention, wellness, and coping strategies.
- **c2.3. Peer support centers**: Operating centers that offer a variety of peer support services, including drop-in support, peer respite, workshops, and group meetings.

#### c3. Peer worker partnerships

*Boundary/operational definition*: Collaboration between Peer Workers and professional healthcare providers, integrating peer support into formal care settings.

- **c3.1. Integration with clinical services**: Working alongside clinicians to deliver integrated care, ensuring peer support complements clinical interventions.
- **c3.2. Multi-disciplinary teams**: Participating in teams that include various healthcare providers, offering a holistic approach to recovery support.
- **c3.3. Referral and navigation services**: Assisting peers in accessing additional health or social services, helping them navigate complex systems (e.g., linking patients with SUD in the emergency department to recovery support services) (33).

#### c4. Peer worker-led advocacy

*Boundary/operational definition*: Activities focused on advancing the rights and needs of Peer Workers in recovery, often involving public policy, stigma reduction, and representation.

- **c4.1. Policy advocacy**: Engaging in efforts to influence public policy to better support individuals with SUD and those in recovery.
- **c4.2. Stigma reduction campaigns**: Leading or participating in campaigns aimed at reducing the stigma associated with SUD and recovery.
- **c4.3. Peer worker representation in advisory boards**: Serving on advisory boards or committees to ensure that their voices are included in decision-making processes.

### d. Support settings

PRSS can be delivered in multiple settings within regional service networks and across each stage of change (14, 34, 35). This taxon proposes three overarching macro categories in which most of the micro settings in which PRSS occur can be nested or described.

#### d1. Community-based settings

*Boundary/operational definition*: Non-clinical environments where peer support is provided, often within the community or faith-based organizations.

- **d1.1. Recovery houses and shelters**: Providing peer support in residential environments that offer transitional living for those in recovery.
- **d1.2. Recovery community centers**: Engaging with Peer Workers in free, accessible, Peer Worker-led centers/spaces that are located within the communities they serve, offering recovery support through education and capacity building, social/recreational activities, individual and group peer support, community engagement, and linkage/referral to local community resources.
- **d1.3. Faith-based organizations**: Delivering peer support within religious or spiritual communities, integrating faith with recovery.
- **d1.4. Educational settings**: Delivering support in recovery high schools or prevention programming in public schools.
- **d1.5. Recovery friendly workplaces**: Offering peer support within the economic sector and advocating for fair chance employment and non-stigmatizing drug free workplace policies.
- **d1.6. Harm reduction services**: PRSS can provide services within harm reduction settings such as syringe services programs to support engagement with peers who are currently using substances.
- **d.1.7. Street outreach services**: Street outreach PRSS can support access to immediate basic needs.

#### d2. Clinical settings

*Boundary/operational definition*: Formal healthcare environments where peer support is integrated into medical or therapeutic services.

- **d2.1. Hospitals and emergency rooms**: Providing peer support to individuals in crisis or receiving medical care in hospital settings (36).
- **d2.2. Outpatient clinics**: Supporting Peer Workers in outpatient treatment or specialty care centers (e.g., obstetrics, cancer care, etc.), where they receive ongoing medical or therapeutic care.
- **d2.3. Inpatient rehabilitation facilities**: Offering peer support within residential treatment programs focused on intensive recovery efforts.

#### d3. Justice system settings

*Boundary/operational definition*: Correctional or legal settings where peer support is provided to individuals involved with the legal justice system.

- **d3.1. Drug/recovery courts**: Supporting individuals who are navigating drug court programs as part of their recovery process.
- **d3.2. Prisons and jails**: Offering peer support to incarcerated individuals, helping them prepare for reintegration into the community.
- **d3.3. Reentry programs**: Assisting Peer Workers in transitioning from incarceration back into the community, providing support for successful reintegration.

### e. Support activities

Support activities encompass the range of services provided by Peer Workers, tailored to the support approach, setting, and type of training they have received. Literature describing these activities is still emerging, and PRSS programs often report “recovery support” services without specifying the activities performed (36). Recent research is beginning to delineate these activities based on type, setting, frequency, and recovery phase (32, 35, 37). SAMHSA (38) categorizes recovery support into four areas: emotional, informational, instrumental, and affiliational. Below, we align Peer Worker activities with these categories.

#### e1. Emotional support

*Boundary/operational definition*: Emotional support involves activities where Peer Workers provide psychological and empathetic assistance to help peers manage emotions and challenges in their recovery journey.

- **e1.1. Relationship building**: Establishing trust and rapport to engage peers, catalyzing their engagement with additional services.
- **e1.2. Increasing motivation for change**: Using motivational interviewing and other techniques to build peer self-efficacy.
- **e1.3. Reducing self-stigma and shame**: Helping peers overcome negative self-perceptions and stigma.
- **e1.4. Trauma-informed care and support**: Providing tailored interventions for peers with trauma, including victims of violence or trafficking.
- **e1.5. Active listening and building rapport**: Engaging in empathetic, non-judgmental listening to help peers express themselves.
- **e1.6. Encouragement and inspiring hope**: Offering positive reinforcement, often through storytelling, to build self-esteem and demonstrate that recovery is achievable.
- **e1.7. Accompaniment**: Supporting peers in accessing services where they may feel stigmatized or hesitant.

#### e2. Informational support

*Boundary/operational definition*: Informational support focuses on educating and empowering peers by providing information, skills, and resources that facilitate informed decision-making and enhance recovery management.

- **e2.1. Psychoeducation**: Educating peers about mental health, SUD, and treatment options, promoting informed recovery decisions.
- **e2.2. Skill-building**: Offering life skills training, financial literacy education, coping strategies, and health and wellness practices.
- **e2.3. Health literacy**: Assisting peers in understanding health conditions, treatment options, and navigating the healthcare system.
- **e2.4. Recovery coaching**: Structured guidance focusing on achieving and maintaining recovery milestones.
- **e2.5. Recurrence prevention support**: Identifying and managing triggers to prevent substance use recurrence.
- **e2.6. Crisis de-escalation**: Assisting in managing emotional crises to prevent escalation.

#### e3. Instrumental support

*Boundary/operational definition*: Instrumental support involves helping peers access tangible resources, advocating on their behalf, and reducing systemic barriers that may impede recovery.

- **e3.1. Resource navigation**: Helping peers access housing, employment, and healthcare.
- **e3.2. Screening and referral**: Screening peers for SUD and connecting them to appropriate treatment services.
- **e3.3. Treatment entry assistance**: Supporting peers during treatment initiation, such as through “warm-handoff” programs.
- **e3.4. Harm reduction supply distribution**: Providing supplies (e.g., syringes, naloxone, pipes) to peers at risk of overdose as a harm reduction measure.
- **e3.5. Assertive community outreach**: Engaging underserved or high-risk populations in community or harm reduction settings.
- **e3.6. System navigation**: Assisting peers in accessing and navigating healthcare, legal, and social services.
- **e3.7. Advocacy**: Promoting non-pejorative language and advocating for peers facing stigma, especially within clinical settings (39).
- **e3.8. Forensic or court support**: Providing support to individuals involved in the criminal-legal system (40).

#### e4. Affiliational support

*Boundary/operational definition*: Affiliational support activities aim to connect peers with recovery communities and foster social relationships that are conducive to long-term recovery and well-being.

- **e4.1. Connecting to recovery communities**: Assisting peers in building social support within recovery networks.
- **e4.2. Encouraging recreational activities**: Promoting substance-free recreational and social engagement.
- **e4.3. Social skills development**: Supporting peers in building new social relationships to replace those linked to substance use.
- **e4.4. Long-term mentorship**: Providing ongoing mentorship to sustain long-term recovery, fostering stable, supportive relationships.

### f. Recovery milestones

Recovery milestones refer to recovery-related biopsychosocial outcomes that refer to measurable changes in peers’ health, behavior, and social circumstances that in part result from PRSS interventions. These outcomes are classified as proximal (short-term and directly influenced by PRSS) or distal (long-term and indicative of sustained recovery success). While substance use reduction remains a core milestone in recovery, broader biopsychosocial outcomes—including physical health, mental well-being, housing stability, and employment—serve as critical indicators of long-term recovery. These outcomes reflect established models of recovery capital, which emphasize multidimensional progress rather than a singular focus on substance use. Therefore, this taxonomy integrates biopsychosocial outcomes as essential recovery milestones that align with PRSS goals and holistic recovery frameworks.

#### f1. Proximal outcomes

*Boundary/operational definition*: Proximal outcomes are immediate, short-term effects directly influenced by peer support interventions. These outcomes typically occur within the initial stages of recovery and provide early indications of progress or change as a result of PRSS activities.

- **f1.1. Reduced or no substance use**: Focuses on the reduction in substance use or periods of abstinence. While historically recovery meant total abstinence, contemporary definitions recognize reduction as a dynamic and positive outcome.
- **f1.2. Improved physical health**: Measures enhancements in physical well-being as peers engage in healthier behaviors and access medical support, including medication-assisted treatment (e.g., MAUD, MOUD) for SUD.
- **f1.3. Enhanced mental health**: Evaluates improvements in peers’ mental health status, including reductions in symptoms of anxiety, depression, or other co-occurring disorders and improvements in mental well-being.
- **f1.4. Stable housing**: Focuses on the peer’s ability to secure and maintain stable housing as a component of their recovery.
- **f1.5. Increased self-efficacy**: Assesses the peer’s confidence in their ability to manage recovery and life challenges independently.
- **f1.6. Employment or purposeful daily activities**: Evaluates whether peers have gained employment or engage in meaningful activities that support their recovery.
- **f1.7. Social and community connections/improved relationships**: Examines peers’ ability to form and sustain healthy social and community relationships.
- **f1.8. Reduction criminal justice involvement**: Measures declines in criminal justice involvement and new legal issues as recovery progresses.
- **f1.9. Family reunification**: Assesses improvements in family relationships and reunification efforts, including children, as part of the recovery journey.

#### f2. Distal outcomes

*Boundary/operational definition*: Distal outcomes are long-term effects that reflect the sustained impact of PRSS interventions. These outcomes indicate the overall success of the recovery process, encompassing broader life improvements and stability beyond the initial stages of recovery.

- **f2.1. Long-term resolution of problematic substance use**: The ultimate goal is to achieve and maintain long-term resolution of substance use issues.
- **f2.2. Improved quality of life**: Measures overall improvements in life satisfaction and well-being.
- **f2.3. Economic stability**: Evaluates the peer’s ability to achieve and sustain economic stability through employment and financial management.
- **f2.4. Family and parenting stability**: Assesses long-term family functioning, including parenting stability and the re-establishment of supportive family networks.
- **f2.5. Sustained engagement in SUD treatment and recovery support services**: Peer Workers help peers remain engaged in medication-assisted treatment (e.g., MAUD, MOUD) for SUD, mental and behavioral health supports (e.g., counseling, psychotherapy, medication management), and other medical and behavioral health services.

## Proposed evaluation framework applying the PRSS taxonomy

To establish an empirical evaluation model for PRSS, future studies must integrate independent, dependent, mediator, and moderator variables. The variables identified in this taxonomy should be included in designing robust evaluations that translate actionable insights into the mechanisms and conditions making PRSS most effective (e.g., taxonomy components a-f).

In this example, lived experience functions as the core independent variable, representing the diversity of personal histories that Peer Workers bring to their roles. Training moderates this impact, influencing how lived experience translates into effective support based on variations in type, intensity, and certification level. Mediators—encompassing support approaches, settings, and support activities—clarify the pathways through which lived experience and training of Peer Workers influence recovery outcomes in peers.

- **Independent variable: lived experience (Related to taxon a: peer worker lived experience)**. Lived experience serves as the core independent variable. Peer Workers may possess SUD direct experience or indirect experience, such as supporting family members or working within recovery communities. They may also have experiences with different substances, methods of substance use, houselessness, or specific systems involvement such as child welfare. Differentiating between these types of lived experience, as outlined in taxon a, allows for a deeper understanding of how personal history influences the effectiveness of PRSS delivery.
- **Moderators: training (related to taxon b: peer worker training)**. Training acts as a moderator by influencing how lived experience translates into effective peer support. Training can vary by type (e.g., trauma-informed care, mental health support), intensity (e.g., short workshops versus long-term certification programs), and formal degree/certification levels (e.g., basic peer support certification versus advanced clinical certifications). By including training as a moderator, studies can explore conditions under which specific types or intensities of training enhance or diminish the impact of lived experience, directly aligning with the variations described in taxon b.
- **Mediators: approach/settings/support activities (related to taxons c, d, e: approach, setting, support)**. Mediators explain the pathways through which lived experience and training impact recovery outcomes. They may include the specific approaches used (e.g., one-on-one mentoring, group facilitation) from taxon c, the settings in which PRSS is delivered (e.g., hospitals, recovery centers, community spaces) as described in taxon d, or the specific support activities (e.g., referral services, crisis management) as described in taxon e. Understanding these mediators, and the “mechanism of action” through which they impact peers, helps to clarify how and why peer support leads to specific recovery milestones. For example, through various support approaches, settings, and activities, Peer Workers may influence peers’ recovery journeys by reducing internalized stigma, increasing self-efficacy, or fostering other positive biopsychosocial outcomes, such as sustained use of medication for opioid use disorder (MOUD). While these mechanisms of action provide valuable insights, they are yet to be systematically investigated, leaving critical gaps in understanding how specific support activities translate into measurable recovery outcomes. For example, activities like storytelling and one-on-one mentorship may foster self-efficacy or diminish stigma in different ways (9), depending on the Peer Worker’s lived experience, training, and delivery setting.
  Recognizing mechanisms of action as mediators in the PRSS framework provides a practical lens for examining how specific support activities impact recovery milestones, underscoring the importance of identifying which mechanisms are most effective under specific conditions. This approach clarifies the taxonomy’s categories—support activities (taxon e), approach (taxon c), and setting (taxon d)—as interconnected elements that together create pathways toward recovery.
- **Dependent variables: recovery milestones (related to taxon f: recovery milestones)**. The model evaluates the impact of PRSS on key recovery milestones, which are the dependent variables. By systematically measuring these milestones, researchers can assess the effectiveness of PRSS interventions across diverse peer groups, as aligned with the outcomes detailed in taxon e.

### Application of the model

This proposed evaluation framework allows for testing multiple hypotheses that inform the optimization of PRSS for different subgroups within the recovery population. Examples include:

- **Hypothesis example 1**: Peer Workers with direct lived experience and advanced clinical training may be more effective in addressing complex cases, such as managing and sustaining MOUD adherence, compared to those with basic training. This hypothesis tests the interaction between lived experience and training as a moderator, demonstrating how training levels influence recovery outcomes, supporting the detailed components of taxons a and b.
- **Hypothesis example 2**: The effectiveness of PRSS may vary based on the settings in which they are delivered. For instance, peer support in emergency room settings may demonstrate different outcomes compared to support provided in long-term residential facilities. By examining approach and setting as mediators (taxons c and d), the model reveals how these factors shape the pathway through which lived experience and training translate into recovery milestones.

### Implications

By incorporating these elements, the evaluation framework clarifies pathways through which PRSS impacts recovery milestones, offering insights to optimize services. It highlights that PRSS effectiveness is not uniform but may vary with specific conditions, such as types of lived experience and training levels. This understanding supports tailored interventions that align Peer Worker approaches with peers’ unique needs and contexts.

## Results and discussion

The taxonomy presented here guides the continued evolution of PRSS, ensuring that these services remain effective and responsive to the diverse needs of individuals in recovery. By systematically categorizing the key elements of PRSS—such as lived experience, training, and various support approaches—this taxonomy provides a clear understanding of how these aspects influence recovery milestones, including treatment outcomes like MOUD adherence, substance use reduction, and abstinence. Through this structured approach, the taxonomy aims to enhance the quality and consistency of peer support interventions in SUD treatment by identifying variables which should be included in evaluations of PRSS effectiveness. Understanding the conditions and factors—such as lived experience, training intensity, and service settings—that most significantly influence outcomes will aid in the development of tailored, evidence-based interventions, adaptable to diverse populations and recovery trajectories. Notably, this taxonomy builds upon prior frameworks, such as SAMHSA’s guidelines, by systematically classifying PRSS components into empirically driven taxons and branches. Unlike previous models, this framework explicitly integrates training variations, different forms of lived experience, and mechanisms of action into a structured evaluation model. This novel approach enables targeted research on PRSS effectiveness while offering a practical tool for implementation and policy development.

### Recommendations and future directions

#### Enhancing PRSS practices and interventions

*Tailoring PRSS interventions*: Based on the insights provided by the taxonomy, PRSS programs should adopt a more personalized approach, aligning peer support methods and settings with the specific needs and recovery trajectories of individuals. For instance, Peer Workers with advanced training and specialized skills could be deployed in complex cases, such as individuals requiring support for sustained MOUD use or those transitioning from criminal justice involvement.

*Training and certification optimization*: States and organizations could use the taxonomy to refine their Peer Worker training programs, ensuring that Peer Workers receive not only foundational skills but also specialized training based on the needs of the populations they serve. Incorporating continuous education and supervision components into certification programs can further enhance the effectiveness of Peer Workers. A note of caution: While rigorous training standards are essential for quality, requiring extensive supervision or costly training hours for certification, as noted in SAMHSA’s recent report (2024), may unintentionally limit accessibility and reduce the availability of individuals entering the PRSS workforce.

#### Extending knowledge and application

*Broadening research scope*: The taxonomy provides a foundation for expanding research into various PRSS components. Many aspects of PRSS are not fully delineated; for example, researchers call for further research regarding the different ways Peer Workers are integrated and utilized in various settings (36), the specific activities they undertake, and the traits of Peer Workers and peers that engender favorable interactions (11). Another priority for future investigation pertains to the “mechanisms of action” by which support activities offered by Peer Workers directly affect their clients (32). Such mechanisms are not yet systematically investigated but are essential to understanding whether and how PRSS impact recovery-related biopsychosocial outcomes.

Future studies also need to examine the mediators and moderators identified within the taxonomy to determine how different training levels, approaches, and settings influence recovery outcomes. For example, research could investigate how integrating PRSS into clinical settings like emergency rooms or outpatient clinics impacts treatment adherence and overall recovery milestones.

*Developing standardized measures*: Collaborate with research institutions and practitioners to develop or adapt standardized measures for each variable, enhancing the consistency and comparability of PRSS evaluation studies, ensuring alignment with the components of the taxonomy (a-e). This approach will provide the foundation for consistent and robust meta-analyses across diverse contexts.

#### Adapting PRSS across diverse populations and settings

*Culturally responsive PRSS*: To effectively serve diverse communities, PRSS interventions should be adapted to align with the cultural and social contexts of peers. The taxonomy is flexible enough to allow for adaptations to assess culturally and linguistically specific and responsive care.

*Implementation in non-traditional settings*: Expanding PRSS delivery beyond traditional clinics to non-traditional settings such as housing programs, workplaces, schools, and community centers can increase accessibility and engagement. Pilot studies should test the efficacy of these non-traditional settings, refining the taxonomy as needed to account for unique challenges and opportunities they present.

### Next steps

*Pilot testing*: Begin with pilot studies that apply this evaluation model in various PRSS settings to generate initial data, validate the taxonomy’s relevance, and refine the measurement tools. These studies will assess the taxonomy’s practicality and effectiveness across different types of PRSS contexts and populations. Testing in diverse settings will also highlight areas for improvement, ensuring that the framework remains flexible and relevant across taxons a–f.

*Iterative refinement*: Use feedback from pilot studies and field responses to fine-tune the framework, ensuring scalability and adaptability across different populations and contexts. This flexibility aligns with the taxonomy’s design for responsiveness, allowing adjustments that accommodate the unique needs of various recovery environments.

*Development of standardized measures*: Collaborate with research institutions and practitioners to develop or adapt standardized measures for each variable. Standardizing these measures will enhance the consistency and comparability of PRSS evaluation studies, ensuring alignment with the taxonomy’s components (a-f) and supporting a common language across studies.

*Controlled studies*: Research addressing potential confounding factors, such as socioeconomic status, resource access, cultural factors, and individual differences in treatment adherence, will be essential, as these factors significantly affect recovery outcomes. Controlling for these factors or including them as covariates will help maintain the accuracy and comprehensiveness of PRSS evaluations. A structured evaluation model rooted in a shared taxonomy will strengthen evidence-based practices, improve PRSS effectiveness, and advance recovery outcomes across diverse communities. The taxonomy also provides a foundation for ongoing empirical validation and iterative refinement across varied populations and settings.

Of note, although this taxonomy was developed within a U.S. context, many of its components—such as peer worker training levels, support approaches, and lived experience classifications—have broad applicability. However, cultural, legal, and healthcare system variations across countries may influence PRSS implementation. Future research should explore how this framework translates to international settings and identify necessary adaptations.

## Conclusion

The proposed taxonomy and evaluation framework advances the PRSS field by systematically categorizing the components and mechanisms that drive effective peer recovery support delivered by Peer Workers. This structured approach guides the development, empirical testing, and refinement of tailored, evidence-based interventions while establishing a shared language for researchers. With this common terminology, researchers can evaluate, compare, and replicate findings consistently across studies, fostering collaboration and knowledge-building within the PRSS research and clinical communities.

We assert, however, that this taxonomy is a foundational framework and is not static. As PRSS research expands and new implementation insights emerge, the taxonomy should evolve through empirical validation, field testing, and practitioner feedback. Future studies should refine taxons and branches as additional evidence accumulates.

In summary, future research should empirically test the taxonomy’s validity through pilot studies, field assessments, and longitudinal tracking of PRSS implementation. Studies require classification of PRSS components in this structured manner to confirm service effectiveness, workforce training quality, and client outcomes. This includes testing the hypotheses generated by the taxonomy, examining mediators and moderators that influence recovery outcomes, and refining its structure as new insights emerge. Ultimately, this framework will generate findings that equip Peer Workers to provide more effective support, enabling individuals in recovery to achieve sustainable progress and an improved quality of life.

## References

[1] Goldstein RZ Volkow ND. Dysfunction of the prefrontal cortex in addiction: neuroimaging findings and clinical implications. Nat Rev Neurosci. (2011) 12:652–69. doi: 10.1038/nrn3119, PMID: 22011681 PMC3462342

[2] McLellan AT Kushner H Metzger D Peters R Smith I Grissom G. The fifth edition of the addiction severity index. J Subst Abus Treat. (1992) 9:199–213. doi: 10.1016/0740-5472(92)90062-s, PMID: 1334156

[3] Spencer M. R. Garnett M. F. Miniño A. M. (2024). Drug overdose deaths in the United States, 2002–2022. 491. Available online at: https://stacks.cdc.gov/view/cdc/135849 (Accessed April 18, 2025).

[4] U.S. Centers for Disease Control and Prevention. (2024). U.S. overdose deaths decrease in 2023, first time since 2018. Available online at: https://www.cdc.gov/nchs/pressroom/nchs_press_releases/2024/20240515.htm (Accessed April 18, 2025).

[5] Peer Recovery Center of Excellence. (2024). National Distribution of certified peer support specialists in the United States by state, district, and territory—peer recovery Center of Excellence. Available online at: https://peerrecoverynow.org/product/national-distribution-of-certified-peer-support-specialists-in-the-united-states-by-state-district-and-territory/ (Accessed April 18, 2025).

[6] Substance Abuse and Mental Health Services Administration (SAMHSA). (2024). Recovery and recovery support. Available online at: https://www.samhsa.gov/find-help/recovery (Accessed April 18, 2025).

[7] Salzer MS Schwenk E Brusilovskiy E. Certified peer specialist roles and activities: results from a national survey. Psychiatric Services (Washington, DC). (2010) 61:520–3. doi: 10.1176/ps.2010.61.5.520, PMID: 20439376

[8] Eddie D Hoffman L Vilsaint C Abry A Bergman B Hoeppner B. Lived experience in new models of Care for Substance use Disorder: a systematic review of peer recovery support services and recovery coaching. Front Psychol. (2019) 10:1052. doi: 10.3389/fpsyg.2019.01052, PMID: 31263434 PMC6585590

[9] Anvari MS Kleinman MB Massey EC Bradley VD Felton JW Belcher AM. “In their mind, they always felt less than”: the role of peer workers in shifting stigma as a barrier to opioid use disorder treatment retention. J Subst Abus Treat. (2022) 138:108721. doi: 10.1016/j.jsat.2022.108721, PMID: 35067397 PMC9167238

[10] Gagne CA Finch WL Myrick KJ Davis LM. Peer Workers in the Behavioral and Integrated Health Workforce: opportunities and future directions. Am J Prev Med. (2018) 54:S258–66. doi: 10.1016/j.amepre.2018.03.010, PMID: 29779550

[11] Reif S Braude L Lyman DR Dougherty RH Daniels AS Ghose SS. Peer recovery support for individuals with substance use disorders: assessing the evidence. Psychiatric Services (Washington, DC). (2014) 65:853–61. doi: 10.1176/appi.ps.201400047 24838535

[12] White W. (2009). Peer-based addiction recovery support: history, theory, Practice, and scientific evaluation executive summary (pp. 54–59). Great Lakes Addiction Technology Transfer Center.

[13] Gormley MA Pericot-Valverde I Diaz L Coleman A Lancaster J Ortiz E. Effectiveness of peer recovery support services on stages of the opioid use disorder treatment cascade: a systematic review. Drug Alcohol Depend. (2021) 229:109123. doi: 10.1016/j.drugalcdep.2021.109123, PMID: 34700201

[14] Stack E Hildebran C Leichtling G Waddell EN Leahy JM Martin E. Peer recovery support services across the continuum: in community, hospital, corrections, and treatment and recovery agency settings - a narrative review. J Addict Med. (2022) 16:93–100. doi: 10.1097/ADM.0000000000000810, PMID: 33560695 PMC8339174

[15] Ashford RD Curtis B Brown AM. Peer-delivered harm reduction and recovery support services: initial evaluation from a hybrid recovery community drop-in center and syringe exchange program. Harm Reduct J. (2018) 15:52. doi: 10.1186/s12954-018-0258-2, PMID: 30348170 PMC6198436

[16] Chisholm J Petrakis M. Peer worker perspectives on their potential role in the success of implementing recovery-oriented practice in a clinical mental health setting. J Evid Based Soc Work. (2020) 17:300–16. doi: 10.1080/26408066.2020.1729282, PMID: 32420832

[17] Cronise R Teixeira C Rogers ES Harrington S. The peer support workforce: results of a national survey. Psychiatr Rehabil J. (2016) 39:211–21. doi: 10.1037/prj0000222, PMID: 27618458

[18] Hogue A Satcher MF Drazdowski TK Hagaman A Hibbard PF Sheidow AJ. Linkage facilitation services for opioid use disorder: taxonomy of facilitation practitioners, goals, and activities. J Substance Use and Addiction Treatment. (2024) 157:209217. doi: 10.1016/j.josat.2023.209217, PMID: 37981242 PMC10922806

[19] Tong A Sainsbury P Craig J. Consolidated criteria for reporting qualitative research (COREQ): a 32-item checklist for interviews and focus groups. Int J Qual Health Care. (2007) 19:349–57. doi: 10.1093/intqhc/mzm042, PMID: 17872937

[20] Hasson F Keeney S McKenna H. Research guidelines for the Delphi survey technique. J Adv Nurs. (2000) 32:1008–15. doi: 10.1046/j.1365-2648.2000.t01-1-01567.x 11095242

[21] Hsu C-C Sandford B. The Delphi technique: making sense of consensus. Pract Assess Res Eval. (2007) 12:1–8.

[22] Substance Abuse and Mental Health Services Administration. National Model Standards for peer support certification (no. publication no. PEP23-10-01-001) Office of Recovery, Substance Abuse and Mental Health Services Administration (2023).

[23] Kirk MR Dawkins AD Wei X Ajumobi O Lee LC Oman R. What makes a peer? Characteristics of certified peer recovery support specialists in an emergency department-based intervention. PLoS One. (2023) 18:e0289920. doi: 10.1371/journal.pone.0289920, PMID: 38060503 PMC10703250

[24] Kelly JF Abry AW Fallah-Sohy N. Mutual help and peer support models for opioid use disorder recovery In: Kelly JF Wakeman SE, editors. Treating opioid addiction: Springer International Publishing (2019). 139–67.

[25] Scannell C. By helping others we help ourselves: insights from peer support workers in substance use recovery. Adv Ment Health. (2022) 20:232–41. doi: 10.1080/18387357.2021.1995452

[26] Substance Abuse and Mental Health Services Administration (SAMHSA). (2023). 2022 National Survey on drug use and health (NSDUH) releases. Available online at: https://www.samhsa.gov/data/release/2022-national-survey-drug-use-and-health-nsduh-releases (Accessed April 18, 2025).

[27] Substance Abuse and Mental Health Services Administration. (2015). Core competencies for peer Workers in Behavioral Health Services. Available online at: https://www.samhsa.gov/sites/default/files/programs_campaigns/brss_tacs/core-competencies_508_12_13_18.pdf (Accessed April 18, 2025).

[28] Adjabeng BK de Saxe Zerden L. Assessing the training for certified peer support specialists who provide mental health and substance use services. J Behav Health Serv Res. (2024) 51:338–54. doi: 10.1007/s11414-024-09879-2, PMID: 38847957

[29] Felton JW Abidogun TM Senters K Maschino LD Montgomery BW Tyson R. Peer recovery coaches perceptions of their work and their implications for training, support and personal recovery. Community Ment Health J. (2023) 59:962–71. doi: 10.1007/s10597-022-01080-z, PMID: 36595145

[30] Jivanjee P Grover L Thorp K Masselli B Bergan J Brennan EM. Training needs of peer and non-peer transition service providers: results of a National Survey. J Behav Health Serv Res. (2020) 47:4–20. doi: 10.1007/s11414-019-09667-3, PMID: 31240441

[31] Mumba MN Sweeney A Jennings C Matthews J Andrabi M Hall J. Perceived educational needs of substance use peer support specialists: a qualitative study. Community Ment Health J. (2024) 60:160–8. doi: 10.1007/s10597-023-01176-0, PMID: 37606851

[32] Pantridge CE Charles VA DeHart DD Iachini AL Seay KD Clone S. A qualitative study of the role of peer support specialists in substance use disorder treatment: examining the types of support provided. Alcohol Treat Q. (2016) 34:337–53. doi: 10.1080/07347324.2016.1182815

[33] Carpenter J Ibragimov U Steck A Getz T Li Y Giordano N. Implementing peer recovery coaches to increase linkages to recovery services among patients with substance use disorders seen in emergency departments. Emergency Med J: EMJ. (2024). 9, 199–213. doi: 10.1136/emermed-2023-213700 PMC11581905 39209517

[34] Castedo de Martell S Wilkerson JM Howell J Brown HS Ranjit N Holleran Steiker L. The peer to career pipeline: an observational study of peer worker trainee characteristics and training completion likelihood. J Substance Use and Addiction Treatment. (2024) 159:209287. doi: 10.1016/j.josat.2023.209287, PMID: 38160878 PMC10947928

[35] Hagaman A Foster K Kidd M Pack R. An examination of peer recovery support specialist work roles and activities within the recovery ecosystems of central Appalachia. Addict Res Theory. (2023) 31:328–34. doi: 10.1080/16066359.2022.2163387, PMID: 40101104

[36] Jennings LK Lander L Lawdahl T McClure EA Moreland A McCauley JL. Characterization of peer support services for substance use disorders in 11 US emergency departments in 2020: findings from a NIDA clinical trials network site selection process. Addict Sci Clin Pract. (2024) 19:26. doi: 10.1186/s13722-024-00453-x, PMID: 38589934 PMC11003047

[37] Hagaman A. (2021). Peer recovery support specialists: Role clarification and fit within the recovery ecosystems of central Appalachia [Ph.D., East Tennessee State University].

[38] Center for Substance Abuse Treatment. What are peer recovery support services? (no. HHS publication no. (SMA) 09–4454) U.S. Department of Health and Human Services (2009).

[39] Slater TM Rodney T Finnell DS. Promoting the integration of peer support specialists into the healthcare team. Nursing. (2023) 53:50. doi: 10.1097/01.NURSE.0000903972.32588.ad 36700816

[40] Adams WE Lincoln AK. Forensic peer specialists: training, employment, and lived experience. Psychiatr Rehabil J. (2020) 43:189–96. doi: 10.1037/prj0000392, PMID: 31621351

